# Sex‐Differences in the Association of 24‐h Blood Pressure Level and Variability with Subclinical Markers of Cerebral Small Vessel Disease

**DOI:** 10.1002/alz.089898

**Published:** 2025-01-09

**Authors:** Shana B Garza, Sokratis Charisis, Dhrumil Patil, Kristina P. Vatcheva, Silvia Mejia‐Arango, Luis Javier Mena, Ney Alliey‐Rodriguez, Claudia L Satizabal, Carlos A Chavez, Ciro Gaona, Egle Silva, Joseph H. Lee, Joseph D. Terwilliger, Jose Gutierrez, Sudha Seshadri, Gladys E. Maestre, Jesus D Melgarejo

**Affiliations:** ^1^ Institute of Neuroscience, Harlingen, TX USA; ^2^ Glenn Biggs Institute for Alzheimer’s & Neurodegenerative Diseases, University of Texas Health Sciences Center at San Antonio, San Antonio, TX USA; ^3^ Beth Israel Deaconess Medical Centre, Harvard Medical School, Boston, MA USA; ^4^ School of Mathematical and Statistical Science, UTRGV, Brownsville, TX USA; ^5^ South Texas Alzheimer’s Disease Research Center, Harlingen, TX USA; ^6^ Institute of Neuroscience at the University of Texas Rio Grande Valley, Harlingen, TX USA; ^7^ Polytechnic University of Sinaloa, Maztlan, Sinaloa Mexico; ^8^ The University of Texas Rio Grande Valley School of Medicine, Harlingen, TX USA; ^9^ RGV Alzheimers Center (AD‐RCMAR), Brownsville, TX USA; ^10^ Glenn Biggs Institute for Alzheimer’s & Neurodegenerative Diseases, University of Texas Health Science Center at San Antonio, San Antonio, TX USA; ^11^ Laboratory of Neuroscience, University of Zulia, Maracaibo Venezuela (Bolivarian Republic of); ^12^ Laboratory of Ambulatory Recordings, Cardiovascular Institute (IECLUZ), University of Zulia, Maracaibo Venezuela (Bolivarian Republic of); ^13^ Columbia University Irving Medical Center, New York, NY USA; ^14^ Taub Institute for Research on Alzheimer’s Disease and the Aging Brain, Columbia University, New York, NY USA; ^15^ Division of Medical Genetics, New York State Psychiatric Institute, New York, NY USA; ^16^ Sergievsky Center & Department of Epidemiology, Columbia University Medical Center, New York, NY USA; ^17^ Division of Public Health Genomics, National Institute for Health and Welfare, Helsinki Finland; ^18^ Vagelos College of Physicians and Surgeons, Columbia University, New York, NY USA; ^19^ Glenn Biggs Institute for Alzheimer’s & Neurodegenerative Diseases, University of Texas Health Science Center, San Antonio, TX USA; ^20^ South Texas Alzheimer’s Disease Research Center, San Antonio, TX USA; ^21^ The University of Texas Rio Grande Valley School of Medicine, Brownsville, TX USA; ^22^ University of Texas Rio Grande Valley School of Medicine, Brownsville, TX USA; ^23^ RGV Alzheimer’s Center (AD‐RCMAR), Brownsville, TX USA

## Abstract

**Background:**

High blood pressure (BP) level and variability has been linked to stroke, cerebral small vessel disease (CSVD), cognitive decline, and dementia. However, it is unclear whether sex/gender influences the association of BP level and variability with CSVD. Therefore, we aimed to examine sex‐differences in the association of CSVD with 24‐h BP level and variability.

**Methods:**

We used 374 participants aged ≥40y from the Maracaibo Aging Study (women, 73.5%; mean age, 59.1y) who underwent 24‐h ambulatory BP monitoring and brain magnetic resonance imaging (MRI) scans. The 24‐h systolic BP variability was quantified as the average real variability (ARV) index. Markers of CSVD were visualized on MRI scans and included log‐transformed white matter hyperintensities (log‐WMH) volume, and the presence (yes/no) of lacunes, cerebral microbleeds (CMB), or enlarged perivascular spaces (EPVS). The association of 24‐h systolic BP level and variability with markers of CSVD was analyzed using adjusted logistic and linear regression separately for men and women.

**Results:**

Overall, men had greater log‐WMH volume (median, 2.9 vs. 2.5 cm^3^) and higher proportion of lacunes (18.2% vs. 8.7%) than women (*P*≤0.011). The proportion of CMB and EPVS was similar between women and men (*P*≥0.522). A higher 24‐h systolic BP level was associated with log‐WMH in men (adjusted β‐coefficient, 0.37; 95% CI 0.08, 0.21) and women (adjusted β‐coefficient, 0.16; 95% CI, 0.01, 0.09) as well as the presence of lacunes in both groups (the OR for men was 2.31 [95% CI, 1.08‐4.95] and for women was 1.55 [95% CI, 1.02‐2.37]). Only in women, a higher 24‐h ARV systolic BP was associated with log‐WMH (adjusted β‐coefficient, 0.19; 95% CI, 0.02, 0.10), lacunes (OR, 1.81; 95% CI, 1.15‐2.84), and EPVS (OR, 1.67; 95% CI, 1.07‐2.60).

**Conclusions:**

We found sex‐differences in the association 24‐h BP level and variability with markers of CSVD. In men, high 24‐h BP level had a greater impact on the burden of WMH and lacunes than in women whereas high 24‐h BP variability seems to be a risk for CSVD only in women. These results suggest the potential influence of sex in the relationship between vascular risk factors and CSVD.

